# Typology Research Questionnaires

**Published:** 1996

**Authors:** Kathryn G. Ingle

**Affiliations:** Kathryn G. Ingle is a science editor of *Alcohol Health & Research World*

**Keywords:** questionnaire, AOD dependence, disorder classification, research and evaluation, method, personality test, alcohol use test, treatment

## Abstract

Alcoholism typology research is concerned with categorizing people based on their genetic risk, drinking patterns, and personal characteristics, among other factors. Thus, scientists in this field rely on tools such as personality questionnaires to determine the category, or subtype, in which each alcoholic subject belongs. Five frequently used questionnaires are reviewed—the Minnesota Multiphasic Personality Inventory, the MacAndrew Alcoholism Scale, the Eysenck Personality Questionnaire, the Tridimensional Personality Questionnaire, and the Connecticut Typology Questionnaire—and some sample questions are shown.

As evidenced by this issue of *Alcohol Health & Research World*, clinicians and researchers are addressing the challenge of matching patients to specific therapies by first defining who alcoholic patients are and what circumstances have caused them to abuse alcohol. Ultimately, this approach should help shape treatment offerings. Although no health care system can possibly tailor its care to meet every treatment need encountered in the alcoholic population, scientists studying this population suggest that it could be divided into groups of people with similar needs. Members of each group could then enter a form of treatment that most closely addresses their group’s set of common problems and thus, presumably, will be the most effective in turning them toward recovery.

Typology research has grown out of the need to establish such groups, or subtypes, of alcoholics for both treatment and research purposes. A typology defines collections of characteristics that often accompany alcoholism but also set one group of people with the disorder apart from another. Researchers can develop typologies in two basic ways: (1) divide the population according to predefined characteristics, such as whether a person has a family history of alcoholism or an alcoholic personality type, or (2) evaluate the population using a variety of tests and identify clusters of characteristics that tend to differentiate groups of people from each other (Babor and Dolinsky 1988). Both methods often require the use of questionnaires that aid in defining patients’ personality factors, alcohol consumption patterns, or family histories of alcohol-related disorders. This article reviews several of the most frequently used questionnaires and provides samples of the questions they contain.

## Minnesota Multiphasic Personality Inventory

The Minnesota Multiphasic Personality Inventory (MMPI) is one of the most widely used personality tests (Groth-Marnat 1990). It was developed in 1940 at the University of Minnesota to aid in assessing adult psychiatric patients and the ways in which their conditions responded to psychotherapy. The current version (the MMPI–2), which was developed during the early 1980’s, includes 567 true-or-false items that measure personality factors associated with various psychiatric conditions. According to the second edition of the *Handbook of Psychological Assessment*, “the overall item content is extremely varied and relates to such areas as general health, occupational interests, preoccupations, morale, phobias, and educational problems” (Groth-Marnat 1990, p. 180). The inventory questions are divided into scales intended to reveal the presence of psychiatric diagnoses. Examples of the scales include depression, hysteria, psychopathic deviate, paranoia, schizophrenia, and social introversion. Each scale is composed of questions that differentiate patients with the condition from normal control subjects; that is, the scales are not intended to be compared with each other. Other scales added during the restandardization that produced the MMPI–2 measure personality traits, such as anxiety and cynicism, as well as health concerns (Groth-Marnat 1990).

Often alcohol researchers use the MMPI as only part of their typological assessment (Allen et al. 1994; Babor et al. 1992). For example, while formulating their type A-type B scheme, Babor and colleagues (1992) used the MMPI to assess psychological adjustment among their subjects as they entered alcoholism treatment. One MMPI subscale, considered by MacAndrew (1965) to measure the impulsivity, pressure for action, and acting-out behavior that leads to alcoholism, proved—in Babor and colleagues’ (1992) research—to be an important discriminator of a subtype characterized by developing alcoholism at an early age and experiencing more severe alcohol-related problems. Other typologies have been based almost entirely on MMPI personality assessments and have resulted in as many as seven or more separate subtypes (for a review, see Morey and Skinner 1986). These schemes, however, have been criticized as having limited usefulness in clinical settings because they do not recommend specific treatment regimens for different subtypes (Rounsaville et al. 1987).

## MacAndrew Alcoholism Scale

MacAndrew derived his MacAndrew Alcoholism Scale (MAC) from the MMPI during the 1960’s in an effort to determine whether “alcoholics are simply neurotics-who-also-happen-to-drink-too-much” or if people with alcoholism constitute a distinct population (MacAndrew 1965, p. 238). Among the 49 MMPI true-false statements that MacAndrew selected to differentiate alcoholic patients from psychiatric patients are the following:

- I have had periods in which I carried on activities without knowing later what I had been doing.
- I sweat very easily even on cool days.
- While in trains, buses, etc., I often talk to strangers.
- I do not like to see women smoke.
- I deserve severe punishment for my sins.
- I am a good mixer.

Using the MAC scale, MacAndrew concluded that differences do exist between alcoholic and psychiatric patients. He asserted that the MAC does not, however, distinguish alcoholics from all other people in society. Instead, it identifies a subset of alcoholics who possess a certain “character orientation” (MacAndrew 1981, p. 605). Studies by other researchers who used the MAC to differentiate alcoholic patients help define this orientation. For example, Svanum and Ehrmann (1992) suggested that alcoholics who score high on the MAC are gregarious drinkers and experience more aggression and legal problems as a result of their drinking. In contrast, low-scoring alcoholics are less outgoing and tend to drink alone, although they suffer alcohol-related consequences as serious as those of high MAC scorers. Reviewing additional MAC scale findings, MacAndrew noted that alcoholics with high MAC scores move “(with ‘boldness’) into the world, albeit in a sometimes rancorous and ill-considered fashion, with little regard for future consequences” (MacAndrew 1981, p. 618). Thus, the MAC may most readily identify a single alcoholic subtype.

## Eysenck Personality Questionnaire

The purpose of the Eysenck Personality Questionnaire (EPQ) is to detect the degree to which a person possesses three basic personality dimensions. Eysenck and Eysenck initially developed the Eysenck Personality Inventory (EPI) in 1964. The EPI, which in itself was an improvement on other personality questionnaires, was intended to measure two personality dimensions: extroversion-introversion (i.e., a measure of someone’s sociability) and neuroticism (i.e., a person’s proneness to becoming anxious under duress) (Rosenthal et al. 1990; Eysenck and Eysenck 1964; Buros 1965). Ultimately, Eysenck and Eysenck revised the EPI to form the EPQ, which was published in 1975, by adding the third dimension—impulsivity, or degree of restraint or tough-mindedness (Rosenthal et al. 1990; Hurlburt et al. 1982).

The 90-question EPQ has sometimes been favored for use in psychological studies because its questions focus less on sickness or specific symptoms than do other questionnaires and therefore provoke less resistance from subjects (Rosenthal et al. 1990). The test distinguishes the personality traits characteristic of alcohol and other drug (AOD) abusers from those of healthy members of a general population, showing the abusers to be more impulsive, introverted, and anxious (Rosenthal et al. 1990; Hurlburt et al. 1982). The questionnaire, however, appears to have been rarely employed as the sole basis for an alcoholism typology. Rather, it has been used (in full or in part) to demonstrate personality differences among AOD abusers who also can be grouped according to criteria other than personality (e.g., cognitive capacity) (Alderdice et al. 1994). For example, one study using the EPQ found that users of stimulants (e.g., cocaine) are generally less anxious but more impulsive than alcoholics (Rosenthal et al. 1990). A second study determined that alcohol abusers scored lower in extroversion than did users of other drugs (O’Connor et al. 1995). Other researchers have detected minor EPQ differences between alcoholics of varying race and gender (Hurlburt et al. 1982, 1984). Finally, one study found that alcoholics who were members of Alcoholics Anonymous (AA) had lower EPQ scores on neuroticism and impulsivity and higher scores on extroversion than those who were not AA members (Hurlburt et al. 1984). Thus, the EPQ is often used in typology research in conjunction with other measures that differentiate groups of alcoholics.

## Tridimensional Personality Questionnaire

As its name suggests, the Tridimensional Personality Questionnaire (TPQ)—like the EPQ—was developed to measure three separate dimensions of personality. Its creator, Dr. Robert Cloninger, hypothesizes that these dimensions may have biological bases (i.e., they may reflect differences in brain systems), may be inherited independently of each other, and may interact to mediate a person’s responses to environmental stimuli, including alcohol. According to Cloninger, “these personality traits distinguish alcoholics with different patterns of behavioral, neurophysiological, and neuropharmacological responses to alcohol” (Cloninger 1987, p. 410). The three dimensions are as follows:

- Novelty seeking, or the motivation to seek new experiences and the degree of exhilaration felt in encountering them
- Harm avoidance, or the motivation to avoid situations or behaviors that may result in punishment
- Reward dependence, or the motivation to repeat situations associated with positive reinforcement.

Using a personality model built on these three factors, Cloninger arrived at two clusters of traits that appear to be associated with alcoholism. He has associated the clusters with his type I-type II typology (for further description of this typology, see the article by Cloninger, pp. 18–23) (Earleywine et al. 1992; Cloninger et al. 1988). Aspects of these clusters can be observed in childhood and may predict the risk for developing alcohol problems. For example, Cloninger has reported that a combination of high novelty seeking and low harm avoidance in children predicts early onset alcohol abuse (Cloninger et al. 1988).

A subject taking the TPQ answers 100 questions designed to assess behaviors typical of each of the three personality dimensions. The test is divided into four subscales for each dimension that delve more specifically into the personality traits making up the dimension. For example, the subscales for novelty seeking are exploratory excitability versus stoic rigidity, impulsiveness versus reflection, extravagance versus reserve, and disorderliness versus regimentation (Earleywine et al. 1992). Alcohol researchers have used the TPQ to study the risk for developing alcoholism, the disorders that co-occur with alcoholism, such as antisocial personality disorder and smoking addiction, and other aspects of the relationship between personality and alcohol use. Some studies, however, have indicated that the TPQ cannot identify personality differences among nonalcoholics with family histories of alcoholism and those with no family histories, as might be expected if the three factors are inherited and related to alcoholism (Hesselbrock and Hesselbrock 1992; Zaninelli et al. 1992; Schuckit et al. 1990).

## The Connecticut Typology Questionnaire

Babor and colleagues developed their A–B typology by using a broad-based battery of clinical assessment tools. Included in this assessment were measures of a family history of alcoholism and other risk factors for alcohol problems, severity of dependence and related problems, co-occurring psychiatric problems, and the length of time a person has been alcoholic. The resulting scheme divided the alcoholic population into two groups: those people with a generally less severe disorder (i.e., type A) and those experiencing more extreme symptoms in each drinking-related category (i.e., type B) (Brown et al. 1994). Babor and colleagues developed the Connecticut Typology Questionnaire from several sources during the 1980’s in order to test subjects on all of the aspects of drinking-related disorders listed above.

The questionnaire comprises an array of tests that ask subjects to report on childhood problem behaviors (e.g., whether subjects tended to lie or get into fights), medical problems, history of and reasons for engaging in AOD abuse, and drinking-related symptoms and problems. It also includes the MAC scale previously discussed. Most questions not included in the MAC scale or in the personal history sections ask how often a statement accurately pertains to the subject and offer several possible answers, ranging from “frequently” to “never” (see figure). Babor and colleagues designed the Connecticut Typology Questionnaire for both research and clinical use. Several ongoing studies currently employ the instrument to observe whether type A and type B alcoholics differ in their responses to various psychotherapy and pharmacotherapy regimens such as the use of the drug naltrexone.

**Figure:**
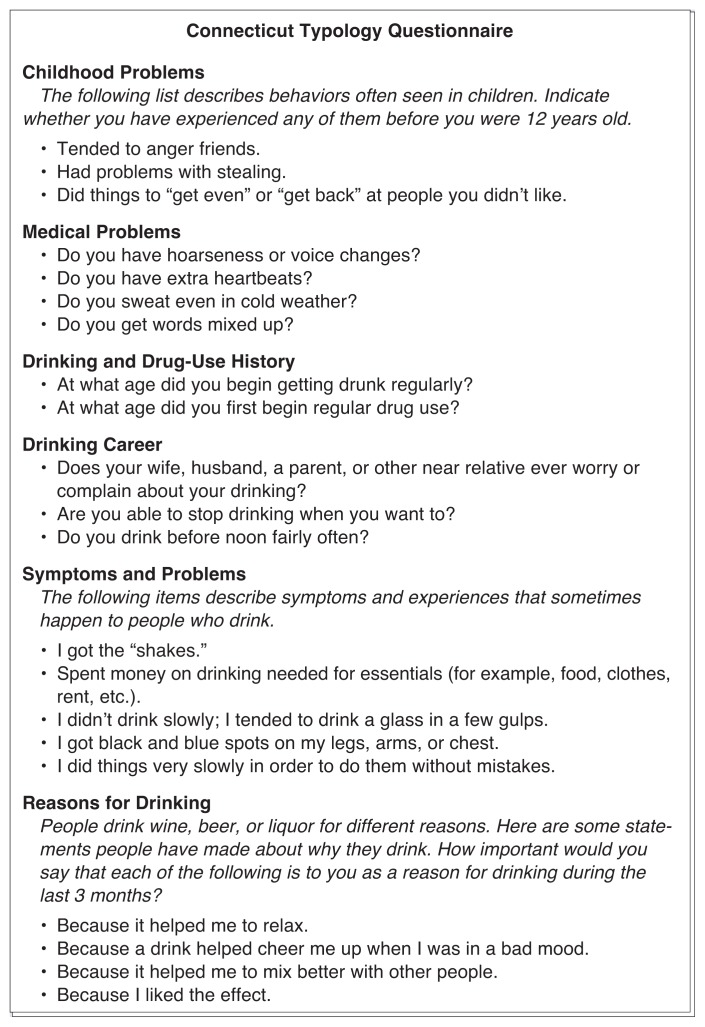
Sample questions from six of the nine sections of the Connecticut Typology Questionnaire. The instructions given vary with each section, from choosing between “never,” “sometimes,” and “frequently” to deciding whether the statement or question applies to the subject’s situation or behavior. SOURCE: Provided by Dr. Thomas F. Babor.

The Connecticut Typology Questionnaire represents the beginnings of the next important phase of typology research—that is, applying the work to a clinical setting. Using clinically effective typologies, clinicians ultimately should be able to match their patients to therapy regimens that best meet the patients’ needs.
